# Deeper and stronger North Atlantic Gyre during the Last Glacial Maximum

**DOI:** 10.1038/s41586-024-07655-y

**Published:** 2024-07-10

**Authors:** Jack H. Wharton, Martin Renoult, Geoffrey Gebbie, Lloyd D. Keigwin, Thomas M. Marchitto, Mark A. Maslin, Delia W. Oppo, David J. R. Thornalley

**Affiliations:** 1 Department of Geography, University College London, London, UK; 2 Department of Geological Sciences, Stockholm University, Stockholm, Sweden; 3 Woods Hole Oceanographic Institution, Woods Hole, MA USA; 4 Department of Geological Sciences and INSTAAR, University of Colorado, Boulder, CO USA

**Keywords:** Palaeoceanography, Physical oceanography, Palaeoclimate

## Abstract

Subtropical gyre (STG) depth and strength are controlled by wind stress curl and surface buoyancy forcing^1,2^. Modern hydrographic data reveal that the STG extends to a depth of about 1 km in the Northwest Atlantic, with its maximum depth defined by the base of the subtropical thermocline. Despite the likelihood of greater wind stress curl and surface buoyancy loss during the Last Glacial Maximum (LGM)^3^, previous work suggests minimal change in the depth of the glacial STG^4^. Here we show a sharp glacial water mass boundary between 33° N and 36° N extending down to between 2.0 and 2.5 km—approximately 1 km deeper than today. Our findings arise from benthic foraminiferal δ^18^O profiles from sediment cores in two depth transects at Cape Hatteras (36–39° N) and Blake Outer Ridge (29–34° N) in the Northwest Atlantic. This result suggests that the STG, including the Gulf Stream, was deeper and stronger during the LGM than at present, which we attribute to increased glacial wind stress curl, as supported by climate model simulations, as well as greater glacial production of denser subtropical mode waters (STMWs). Our data suggest (1) that subtropical waters probably contributed to the geochemical signature of what is conventionally identified as Glacial North Atlantic Intermediate Water (GNAIW)^5–7^ and (2) the STG helped sustain continued buoyancy loss, water mass conversion and northwards meridional heat transport (MHT) in the glacial North Atlantic.

## Abstract (web summary)

Analysis of benthic foraminiferal δ^18^O profiles from sediment cores in two depth transects in the Northwest Atlantic suggests that the subtropical gyre was deeper and stronger during the Last Glacial Maximum compared with today.

## Main

Modern North Atlantic circulation is characterized by two main interconnected systems: first, the basin-wide Atlantic Meridional Overturning Circulation (AMOC), which is a complex system of surface and deep ocean currents that results in vertical overturning. The upper cell of the AMOC transports warm and salty surface and thermocline waters northwards into the high-latitude North Atlantic, in which these waters undergo intense cooling, causing buoyancy loss and subsequent sinking. The resultant dense water masses, known collectively as North Atlantic Deep Water (NADW), flow southwards at depth (1–4 km), in part through the Deep Western Boundary Current (DWBC), which primarily follows the contours of the eastern American continental slope^8,9^. Second, the North Atlantic is also characterized by horizontal, predominantly wind-driven, gyre circulation, composed of an anticyclonic STG and the quasi-cyclonic subpolar gyre (SPG)^10^, both of which also form part of the upper AMOC cell. For example, in the subtropical North Atlantic, upper AMOC flow is carried by the western boundary current of the North Atlantic STG that includes the Gulf Stream, which flows northwards from the tropics along the east coast of North America, eventually separating from the coast at Cape Hatteras^11^. Continuing work is focused on better understanding how these two main circulation systems (the vertical overturning and horizontal gyres) are related in the modern ocean^12^. This includes a recognition that the STG circulation is a substantial contributor to the AMOC and its attendant northwards heat transport, brought about by low-latitude buoyancy loss and STMW formation^13–15^.

Many previous studies have explored how North Atlantic circulation may have altered under fundamentally different past climate states such as the LGM (19–23 thousand years ago (ka))^7,16–20^, when global mean temperatures were roughly 6 °C cooler^21^, ice sheets were at their maximum extent^22^ and sea levels were approximately 120–135 m lower than today^23^. However, there are few glacial data from the Northwest Atlantic, in which both the Gulf Stream and the DWBC transit and a sharp boundary between the STG and SPG is located; thus, hydrographic reconstructions from this crucial transition region provide an opportunity to investigate both vertical and horizontal components of Atlantic circulation during the LGM. Here we do this by generating new profiles of stable oxygen isotope data from two depth transects located at Cape Hatteras–Hudson Canyon Levee (henceforth Cape Hatteras) and Blake Outer Ridge.

**Fig. 1:**
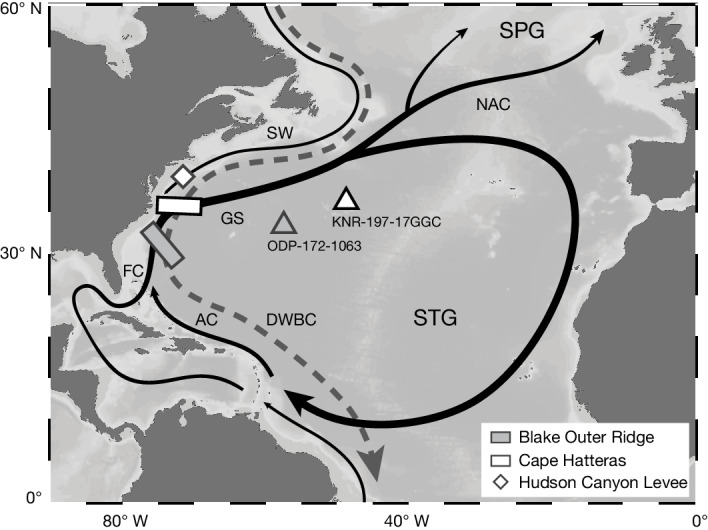
Oceanographic and geographic setting of marine sediment cores used in this study. The solid black and dashed grey arrows denote the main surface and deep ocean currents, respectively. The abbreviations refer to the following currents: AC, Antilles Current; DWBC, Deep Western Boundary Current; FC, Florida Current; GS, Gulf Stream; NAC, North Atlantic Current; SW, Slope Water Current. Ocean bathymetry is based on ETOPO1 global relief data^45^ and was accessed and plotted using Ocean Data View^46^.

Depth transects at Cape Hatteras and Blake Outer Ridge are formed of 10 and 11 marine sediment cores, respectively, and span between about 0.6 and 5.0 km water depth (Fig. 1 and Extended Data Table 1). Today, both transects are bathed by the DWBC below about 1 km (ref. ^24^), but at shallower depths, the STG is more influential at Blake Outer Ridge. By comparison, cores from above 1 km in the Cape Hatteras transect are situated inshore of the Gulf Stream detachment point and as far north as Hudson Canyon Levee (Methods), thus they are predominantly bathed by southwards flowing slope waters of subpolar origin that occupy the upper ocean inshore of the Gulf Stream^25^. Because there was no change in the latitude of the Gulf Stream detachment point during the LGM^17^, the position of the transects relative to the STG/SPG boundary is unchanged from today. Consequently, these transects are ideally located to monitor notable subsurface circulation changes in both the subtropical and the subpolar Northwest Atlantic.

We measured oxygen isotope ratios (δ^18^O = ((^18^O/^16^O)_sample_/(^18^O/^16^O)_standard_ − 1) × 1,000) in benthic foraminifera from sediments corresponding to the mid-to-late Holocene (2–6 ka) and the LGM (Methods), averaging these data to derive mid-to-late Holocene and LGM δ^18^O means for each core. Although variations in benthic foraminiferal δ^18^O reflect both changes in calcification temperature and the δ^18^O of seawater, here we make use of δ^18^O only as a conservative water mass tracer^26^ to help constrain the presence and/or absence of different water masses.

## Deeper glacial subtropical gyre

**Fig. 2:**
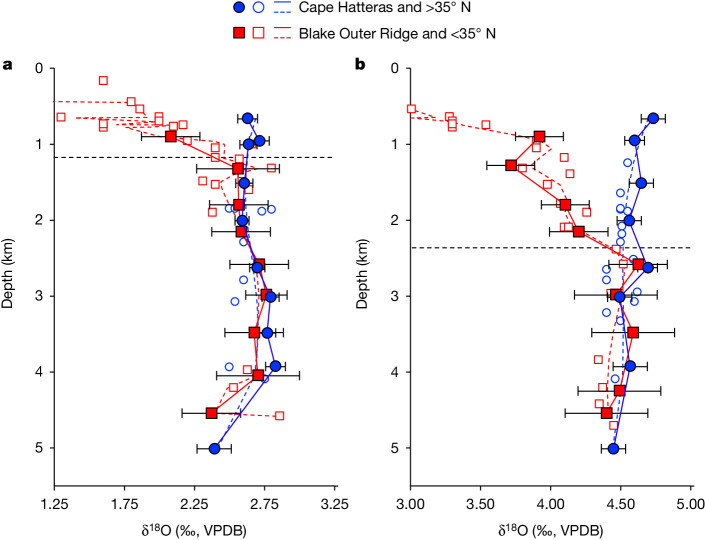
Vertical δ^18^O profiles showing the structure of the ocean at Cape Hatteras and Blake Outer Ridge during the mid-to-late Holocene and LGM. **a**, Mid-to-late Holocene. **b**, LGM. Filled coloured circles and squares represent the mean value for each depth at Cape Hatteras and Blake Outer Ridge, respectively (individual monospecific batch data are shown in Extended Data Fig. 4). Associated error bars are ±2 standard errors (standard error = *σ*/√*n*, in which *σ* is the multispecies replicate error at either Cape Hatteras or Blake Outer Ridge across both time periods and *n* is the number of species analysed at each depth). Open symbols correspond to published δ^18^O from proximal sites in the Northwest Atlantic >35° N (blue circles) and <35° N (red squares; Methods and Source Data)^7,28,47,48^ and dashed coloured lines are smoothing splines through both new and published data >35° N (blue) and <35° N (red). All δ^18^O are reported relative to the VPDB standard. The dashed horizontal black line in each panel denotes the changepoint at which both profiles first converge within error, which we interpret as the maximum depth of the STG. This was done through application of a piecewise linear regression model with one changepoint for all Blake Outer Ridge data from >0.6 km. Source Data

**Extended Data Fig. 1:**
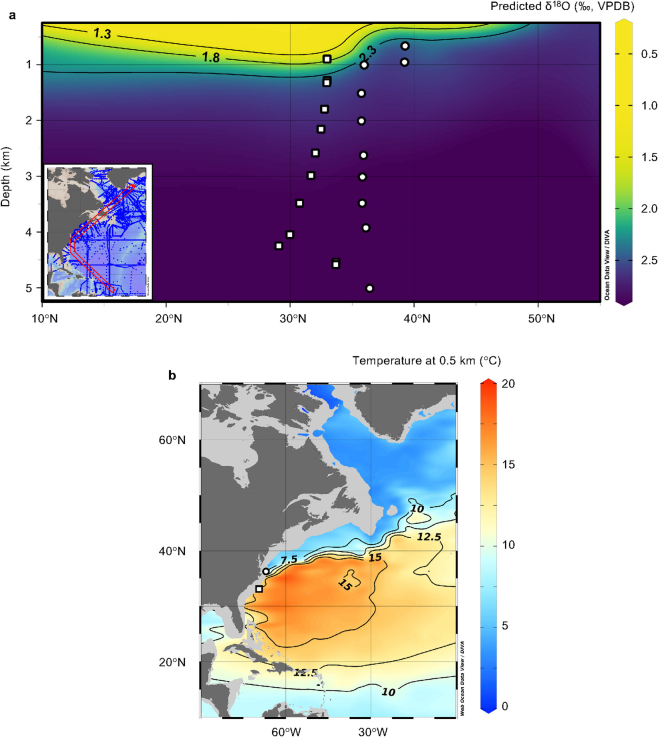
Hydrographic data showing the location of the modern upper ocean subtropical/subpolar boundary at the western margin of the North Atlantic. **a**, Meridional section showing the modern distribution of δ^18^O_calcite_ in the Northwest Atlantic today. Predicted δ^18^O_calcite_ was calculated using temperature and salinity measurements from the Global Ocean Data Analysis Project (GLODAP v2.2022)^60^, empirical calibrations^62^ and the modern North Atlantic (NATL) salinity–δ^18^O_seawater_ relationship^63^, and plotted using Ocean Data View^46^. Open symbols show the locations of sediment cores used in this study. The inset shows the location of stations (blue dots) and section (red box) used to derive the meridional section (**a**). **b**, North Atlantic subsurface temperatures (0.5 km) highlighting the position of the subtropical/subpolar boundary, which is located at about 35° N along the western margin of the basin. Open symbols denote the relative positions of the onshore Cape Hatteras (circles) and Blake Outer Ridge (squares) sites, with respect to the subtropical/subpolar boundary today. Subsurface temperature data are from the GLODAP^60^ and were also plotted using Ocean Data View^46^.

Mid-to-late Holocene δ^18^O profiles from Cape Hatteras and Blake Outer Ridge are very similar below about 1 km (Fig. 2a), probably reflecting the presence of the same deep ocean water mass at both transects during the mid-to-late Holocene, that is, NADW, as is also the case today. (δ^18^O at each core site show good agreement with predicted δ^18^O derived from nearby hydrographic data as well (Extended Data Fig. 4)). By contrast, although δ^18^O from sites shallower than 1.3 km at Cape Hatteras are comparable with deeper sites, δ^18^O from equivalent depths at Blake Outer Ridge decrease with decreasing depth (a trend that is more fully resolved by including published δ^18^O from nearby Northwest Atlantic sites). Today, this upper ocean gradient marks the boundary between the STG and slope waters originating in the SPG and is clearly visible in modern hydrographic data (Extended Data Fig. 1).

In comparison with the mid-to-late Holocene, our glacial data show that the strong upper ocean δ^18^O gradient between Cape Hatteras and Blake Outer Ridge deepened to between 2.0 and 2.5 km (Fig. 2b). This is a result of the entire glacial water column at Cape Hatteras being characterized by relatively high δ^18^O of 4.4–4.7‰, whereas sites at Blake Outer Ridge exhibit a clear trend towards lower δ^18^O above about 2.5 km: δ^18^O is 4.5‰ at 2.5 km, 4.2‰ at 2.0 km, before gradually decreasing to roughly 3.8‰ at 1.0 km and then more sharply decreasing at shallower depths. Because a similar but shallower (roughly 1 km) vertical gradient marks the SPG/STG boundary today (and during the mid-to-late Holocene), we interpret our reconstructed gradient as the glacial equivalent of the SPG/STG boundary, with upper ocean sites at Cape Hatteras and Blake Outer Ridge influenced more by the SPG and the STG, respectively. However, because these glacial gradients extend down to 2.0–2.5 km, we conclude that the STG was approximately twice as deep during the LGM, with substantial amounts of Gulf Stream water penetrating as deep as 2.5 km at Blake Outer Ridge. Carbon isotope (δ^13^C) data are also consistent with previous work^27^ showing high δ^13^C (1.0–1.5‰, Vienna Peedee belemnite (VPDB) standard) at Blake Outer Ridge above about 2 km (Extended Data Fig. 5), which allows us to exclude the possibility of low-δ^18^O/low-δ^13^C southern source waters, for example, Antarctic Intermediate Water, driving these differences^28^.

**Fig. 3:**
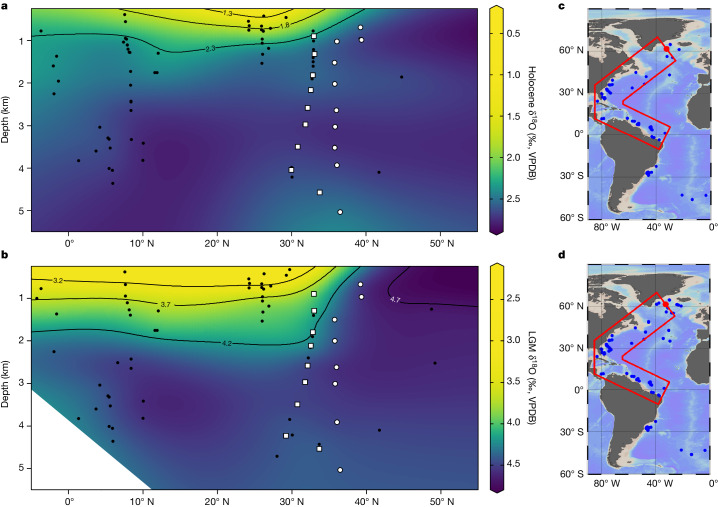
Meridional sections showing the distribution of δ^18^O throughout the Northwest Atlantic during the Holocene and LGM. **a**, Holocene. **b**, LGM. Both sections are derived from a combination of new (open circles) and published^7,28,47–49^ δ^18^O data (filled circles) (Methods and Source Data) from sites located in the red transects in panels **c** and **d**, respectively, and were generated in Ocean Data View^46^ with the Data-Interpolating Variational Analysis (DIVA) tool. Open circles and squares show the relative position of cores in the Cape Hatteras and Blake Outer Ridge transects, respectively, and black dots denote cores with published δ^18^O. Holocene and LGM contours are scaled with an offset of 1.9‰ to account for the approximate shift in δ^18^O_sw_ owing to changes in global ice volume and mean ocean temperatures since the LGM (refs. ^50,51^). Source Data

To place these results in a broader spatial context, we constructed Holocene and glacial δ^18^O sections for the Northwest Atlantic by combining our new data with published δ^18^O data (Fig. 3). The result shows that the locations of our two new glacial transects mark the separation between sites north of 35° N, having δ^18^O higher than or close to 4.5‰ at all depths represented in our profiles, and sites south of 35° N, at which glacial δ^18^O is typically much lower above about 2 km. Furthermore, δ^18^O data from the subtropical western Atlantic reveal that low δ^18^O values, similar to those documented at Blake Outer Ridge, extend meridionally at approximately 2 km depth from roughly 33° N into the equatorial South Atlantic. Thus, published glacial δ^18^O data from the Northwest Atlantic support our interpretation that the STG extended twice as deep during the LGM.

Although previous work has suggested that there was little difference in the depth of the subtropical thermocline between the LGM and today^4^, this was based on a transect of shallow Bahamian cores situated within the on-shelf Gulf Stream, which showed a glacial δ^18^O gradient of about 1‰ extending down through the upper 1 km. However, these data are included here and are consistent with our compilation shown in Fig. 3. Thus, by including data from open-ocean sites, we now show that the enhanced glacial vertical δ^18^O gradient in the subtropics extended as deep as 2.0–2.5 km.

## Thermocline response to glacial forcing

**Fig. 4:**
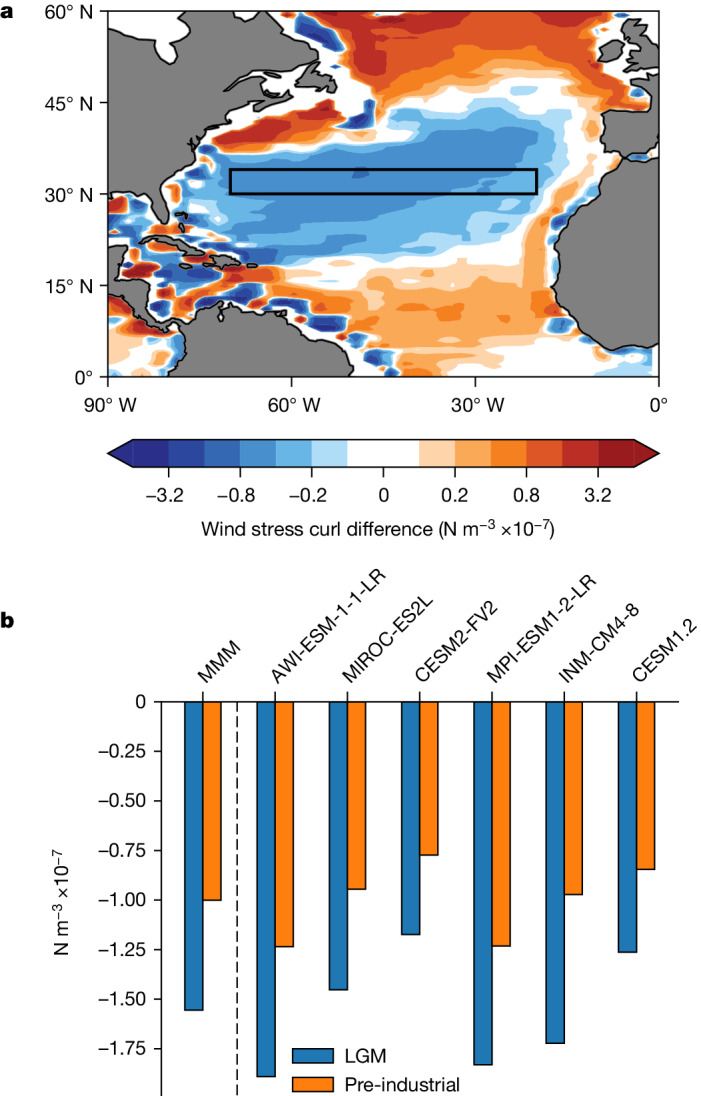
Difference between LGM and pre-industrial wind stress curl over the North Atlantic in PMIP4 climate models. **a**, PMIP4 ensemble mean difference in wind stress curl over the North Atlantic in the LGM relative to the pre-industrial period. **b**, Pre-industrial and LGM mean wind stress curl in the area shown by the black box in **a** for each of the models and the multimodel mean (MMM).

In the modern ocean, the depth of the subtropical thermocline is largely dependent on (1) zonally integrated wind stress curl, which is linearly proportional to the zonal gradient of the depth of the subtropical thermocline, and (2) the depth of the thermocline at its eastern boundary^2^. Therefore, the doubling of STG depth at its western margin that we infer could be explained by changes in integrated wind stress curl and/or changes in eastern boundary conditions. Climate models from the fourth generation of the Paleoclimate Modelling Intercomparison Project (PMIP4) suggest that glacial wind stress curl was approximately 50–80% stronger (mean = 56%, *n* = 6) relative to the pre-industrial period, explaining much of the inferred increase in STG depth (Fig. 4; a similar ensemble mean increase is also seen in PMIP3 models (Extended Data Fig. 6)). The zonal depth gradient of upper ocean isopycnals between 20° W and 74**°** W are also generally steeper in glacial model simulations, consistent with a stronger STG and increased wind stress curl (Extended Data Fig. 7). The remaining portion of the signal could be ascribed to a deepening of the STG along the eastern boundary; however, this is not seen in the model simulations and there is also limited proxy evidence from that region to assess this^29^.

Furthermore, there may have been greater surface buoyancy forcing acting on the STG during the LGM, owing to stronger, cold and dry glacial winds^30,31^. This would have promoted increased heat and buoyancy loss over the Northwest Atlantic and probably led to the generation of denser and deeper STMW^13^. Today, dense STMWs are a well-known feature of the North Atlantic STG, formed near the western boundary of the gyre and its separated extension in the Northeast Atlantic^13,32^. The formation of denser glacial STMWs could also explain the relatively homogenous δ^18^O between about 1 and 2 km depth at Blake Outer Ridge, compared with the steeper gradient above 1 km, with STMWs occupying the water column below 1 km (our δ^18^O profiles suggest that these STMWs were a few degrees warmer than equivalent depths in the subpolar North Atlantic). However, this change in gradient may also reflect the differing properties of subtropical waters circulating on and off the shelf. Notwithstanding, the presence of denser STMWs alongside an intensification of the STG, both driven by glacial atmospheric forcing, are not mutually exclusive processes, thus it is likely that both mechanisms were important in driving a deeper and stronger STG during the LGM.

## Stronger glacial Gulf Stream

Both ventilated thermocline theory and the thermal wind relation predict greater glacial geostrophic velocity at Blake Outer Ridge above about 2 km owing to a deeper and stronger STG^2^. This is consistent with modelled ocean velocities (Extended Data Fig. 8a) and depth transects of sortable silt mean grain size^33,34^, which indicate stronger glacial near-bottom current activity at Blake Outer Ridge core sites shallower than 2.5 km. Notably, no such change is observed further north at Cape Hatteras (Extended Data Fig. 8b), which supports the attribution of this behaviour to the influence of a deeper and stronger glacial Gulf Stream, rather than a possible alternative, such as stronger southwards flowing GNAIW. However, geostrophic transport estimates through the Florida Straits, also based on δ^18^O gradients, were used to infer a weaker Gulf Stream during the LGM^16^. Although this might initially seem contradictory to our inference of a stronger glacial STG circulation, our new glacial data indicate that a substantial proportion of the STG circulation extended to greater depths (>1 km) and thus would have been forced to take offshore, deeper, open-ocean pathways, such as the Antilles Current^31,35^. Lower glacial sea level would also have reduced the proportion of STG circulation through the shelf and Florida Straits^36^. Therefore, we suggest that most of the increase in flow associated with a deeper and stronger STG was through the Antilles Current, bypassing the Florida Straits, and/or the assumption used in the geostrophic method, of a level of no motion at the base of the Florida Straits, was not valid for the LGM. Both scenarios imply that previous glacial Gulf Stream transport estimates based on the geostrophic method at Florida Straits alone are probably an underrepresentation of the STG.

## A revised glacial circulation scheme

Our results provide valuable new mechanistic insights into the structure and circulation patterns of the glacial Northwest Atlantic. For example, nutrient water mass tracers δ^13^C and Cd/Ca have been used to suggest that both the subtropical and the subpolar North Atlantic were largely filled with nutrient-poor, high-δ^13^C (1.0–1.5‰) GNAIW above about 2 km during the LGM^7,37^. However, our new glacial δ^18^O data revealing a sharp upper ocean gradient between subtropical and subpolar waters that extended down to between 2.0 and 2.5 km supports evidence from a modelled glacial inversion that there was a greater contribution of subtropical waters to the glacial deep North Atlantic than in the modern ocean, extending much deeper than today^28^. More importantly, our results provide a dynamic framework for this evidence and further insights into circulation and ventilation of the upper glacial Atlantic. For example, the seemingly homogenous high-δ^13^C water mass bathing the upper 2 km from the equator to 60° N consisted of high-δ^18^O/high-δ^13^C GNAIW north of 36° N, whereas at locations south of 33° N, a large volume of low-δ^18^O/high-δ^13^C subtropical thermocline water was also present. However, one substantial outstanding question concerns the southwards export of GNAIW, which may have been affected by the presence of a deeper STG and the associated increase in subtropical waters down to about 2 km. As well as any entrainment of GNAIW within the STG, and in response to a deeper STG, GNAIW may have: (1) subducted beneath the STG before continuing to flow southwards, (2) taken an eastern pathway around the STG, and/or (3) recirculated northwards as part of the SPG^38–40^. To test these ideas and better constrain glacial circulation will require better spatial coverage of benthic δ^18^O and further proxy reconstructions that can resolve the distinct hydrographic properties associated with different glacial water masses, for example, temperature and δ^18^O_sw_. Furthermore, the observation of a deeper glacial STG forms a key constraint for modelling studies to provide confidence that models accurately simulate the horizontal STG circulation, glacial STMW formation and other aspects of ocean circulation during the LGM.

## Implications for buoyancy and heat fluxes

A deeper and stronger glacial STG also has broader implications for water mass conversion, MHT and biogeochemical cycling. In the modern ocean, the formation of STMW, linked to the STG, results in a strong, shallow overturning cell (when defined in density coordinates^14^) that is an integral part of the AMOC and contributes up to 0.4 PW (about 40%) of the total northwards MHT at 24.0–26.5° N (refs. ^12,13^). Our results imply that more buoyancy loss occurred in the STG during the LGM than in the modern ocean. This may have helped offset the expected reduction in heat-driven buoyancy loss caused by greater sea ice cover and its associated decrease in dense NADW formation in the Nordic Seas^20,41,42^. Moreover, the increase in subtropical buoyancy loss would have helped sustain water mass conversion and northwards MHT in the glacial North Atlantic through buoyancy transformation that was occurring further south than traditional glacial water mass schematics have implied^5,7,37,42^.

Our data also imply a substantial increase in the volume of subtropical water throughout the Northwest Atlantic during the LGM; this may have contributed to nutrient depletion, better oxygenation and high carbonate preservation in the glacial ocean, all of which are thought to contribute to past changes in atmospheric CO_2_ (ref. ^43^). Owing to its role in MHT, STG variability should also be considered when investigating mechanisms of past abrupt climate change, as highlighted in recent idealized model simulations of glacial abrupt climate events^44^. Finally, because the large magnitude changes in STG strength and depth that our proxy data imply are clear targets for testing the accurate representation of the STG in climate models, they can serve to help improve future projections of oceanographic change and their associated impact on marine ecosystems.

## Methods

### Age models

**Extended Data Fig. 2:**
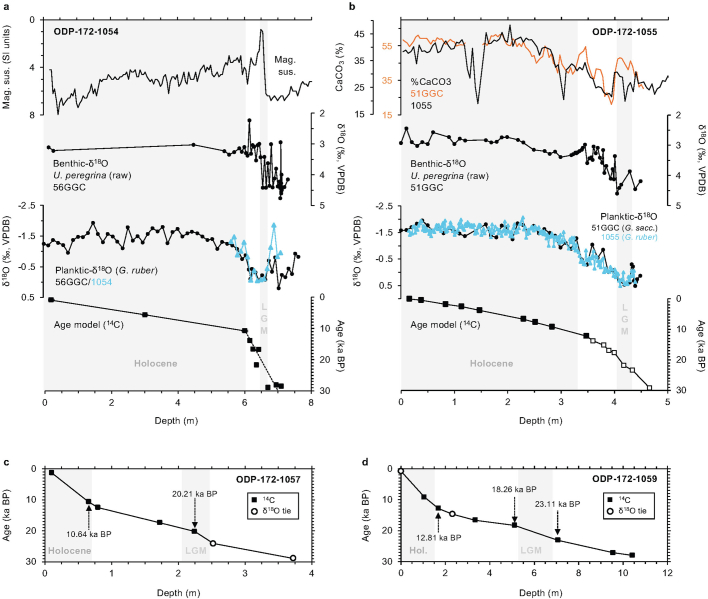
Age models for Blake Outer Ridge transect cores. **a**, Age model for ODP-172-1054 is based on a combination of downcore magnetic susceptibility data and planktic foraminiferal δ^18^O (ref. ^64^), as well as planktic δ^18^O (ref. ^27^) and radiocarbon^65^ from nearby core KNR-140-2-56GGC, the latter of which were transferred on the basis of the alignment of downcore planktic δ^18^O in each core. The resultant LGM section is condensed (about 0.15 m) and thus sensitive to bioturbation; however, single foraminiferal analyses were undertaken to test this possibility (Methods). **b**, Age model for ODP-172-1055 (henceforth 1055) is based on downcore radiocarbon dates^66^ (filled squares) and further dates from nearby core KNR-140-2-51GGC^27^ (open squares), the latter of which were transferred by aligning downcore %CaCO_3_ (ref. ^27^) and planktic δ^18^O (refs. ^27,66^) records from each core. Because glacial sedimentation rates were low, further minor adjustments were also made by aligning the coarse fraction record from 1055 with the NGRIP δ^18^O ice-core record^67^. **c**, Core chronology for ODP-172-1057 is based on planktic foraminiferal radiocarbon and two tie points produced by aligning downcore planktic δ^18^O data with the NGRIP δ^18^O ice-core record (not shown). **d**, Age model for ODP-172-1059 is based on planktic foraminiferal radiocarbon^68^ and two δ^18^O_c_ tie points, which were estimated by aligning downcore planktic δ^18^O with the NGRIP δ^18^O ice-core record and planktic foraminiferal δ^18^O from nearby core KNR-140-2-39GGC^34^. Source Data

**Extended Data Fig. 3:**
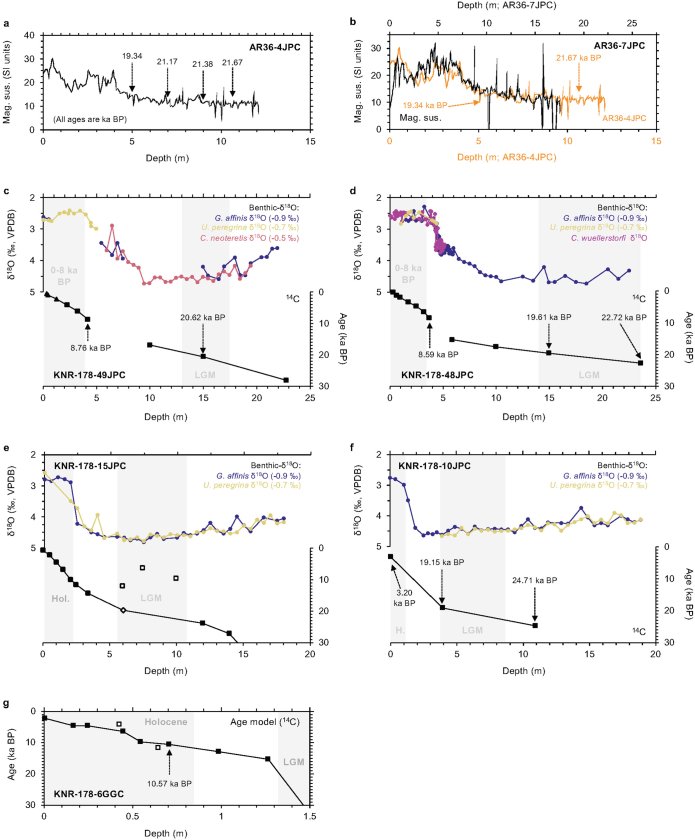
Age models for Cape Hatteras transect cores. **a**, Age model for AR36-4JPC is based on radiocarbon dates on mixed benthic foraminifera. Because the position of the mid-to-late Holocene is unconstrained, benthic δ^18^O in the mid-to-late Holocene transect is sourced from near the core top of companion multicore AR36-MC3C, which has a modern core top. **b**, AR36-7PC is undated, thus the age model is based on the alignment of similar magnetic susceptibility records. Mid-to-late Holocene benthic δ^18^O at this site is from multicore AR36-MC6C, which has a radiocarbon date of 2.86 ka at 30.5 cm. **c**,**d**, Both age models for KNR-178-49JPC and KNR-178-48JPC are based on mixed planktic foraminiferal radiocarbon (black squares and black triangles^69^) and downcore benthic δ^18^O (coloured circles). **e**, Age model for KNR-178-15JPC is based on mixed planktic foraminiferal radiocarbon, downcore benthic δ^18^O and a manual tie point (open diamond) produced by aligning downcore SS data with equivalent data from 49JPC and 48JPC. Open squares indicate rejected young ^14^C dates on mixed planktic foraminifera, reflecting admixture of Holocene planktic foraminifera into glacial-age sediments with much lower planktic foraminiferal abundance. By comparison, benthic δ^18^O is unaffected. **f**,**g**, Both age models for KNR-178-10JPC and KNR-178-6GGC are based on mixed planktic foraminiferal radiocarbon and downcore benthic δ^18^O (open squares indicate rejected dates). Note that KNR-178-6GGC is omitted from the LGM transect owing to bioturbation below about 1 m (abundant burrows were visible). Source Data

New and revised age models for all cores are presented in Extended Data Figs. 2 and 3, with each one based on a combination of downcore benthic foraminiferal δ^18^O, ^14^C, magnetic susceptibility and sediment coarse fraction (>63 μm) data, the first of which typically exhibits low values at depths corresponding to the Holocene and maximum values (within analytical error) during the LGM, indicative of peak glacial conditions and maximum global ice volume. Raw ^14^C dates were converted to calendar years using Marine20 (ref. ^52^) with a Δ*R* correction of −128 ± 68 years (because there is no published open-ocean Δ*R* value from close to either transect, this is a region-specific Δ*R* value, estimated by taking the average of the two nearest sites). In the absence of cores from depths <1 km that contain LGM-age sediments at Cape Hatteras, cores AR36-4JPC and AR36-7JPC from the Hudson Canyon Levee, located along the continental margin about 550 km further north and under the path of the southwards flowing DWBC, are included in the Cape Hatteras LGM transect.

### Core sampling

When possible, cores from Blake Outer Ridge and Cape Hatteras were sampled on the basis of 1,000-year time slices centred at 2.5, 3.5, 4.5 and 5.5 ka for the mid-to-late Holocene and 19.5, 20.5, 21.5 and 22.5 ka for the LGM. A mid-to-late Holocene time slice was chosen because not all Ocean Drilling Program (ODP) cores contain recent sediment. To account for high glacial sedimentation rates (Extended Data Figs. 2 and 3) and sometimes low benthic foraminiferal abundances, large amounts of bulk sediments were required. Therefore, at Blake Outer Ridge, 30 cm of sediment was sampled for each time slice (for example, 19.5 ka), and for core sections with lower sedimentation rates, for example, the LGM in ODP-172-1054, sediments were taken from all depths corresponding to that interval. Because cores from ODP Leg 172 are heavily sampled, samples were sometimes taken from several holes at each core site, with hole suitability evaluated and alignment of different holes achieved by comparing downcore records of physical properties data, for example, magnetic susceptibility and carbonate colour reflectance data. We also used a similar time-slice approach at Cape Hatteras, with each time slice typically consisting of 20-cm sections. For cores in which the temporal extent of the mid-to-late Holocene and LGM is less well defined, we took samples from either side of ^14^C-dated samples, for example, AR36-4JPC.

### Isotopic analyses

Sediment samples corresponding to the LGM and mid-to-late Holocene were weighed, frozen, freeze-dried and disaggregated on a rotating wheel for 24 h before being washed through a 63-μm sieve with deionised water and then oven dried at 40 °C. Monospecific samples of benthic foraminifera were picked from the >212-μm sediment fraction, with samples typically consisting of between five and 15 specimens and weighing between 25 and 200 μg. Depending on sample size, stable oxygen and carbon isotope measurements were performed using a VG Isogas SIRA mass spectrometer with the MultiCarb preparation system (>80 μg) and a Thermo MAT253 isotope ratio mass spectrometer with Kiel IV carbonate preparation device (<80 μg) at the Godwin Laboratory, University of Cambridge. δ^18^O and δ^13^C (δ^13^C = ((^13^C/^12^C)_sample_/(^13^C/^12^C)_standard_ − 1) × 1,000) is reported relative to the VPDB standard, and for both instruments, analytical precision is estimated to be better than ±0.07‰ and ±0.04‰, respectively.

**Extended Data Fig. 4:**
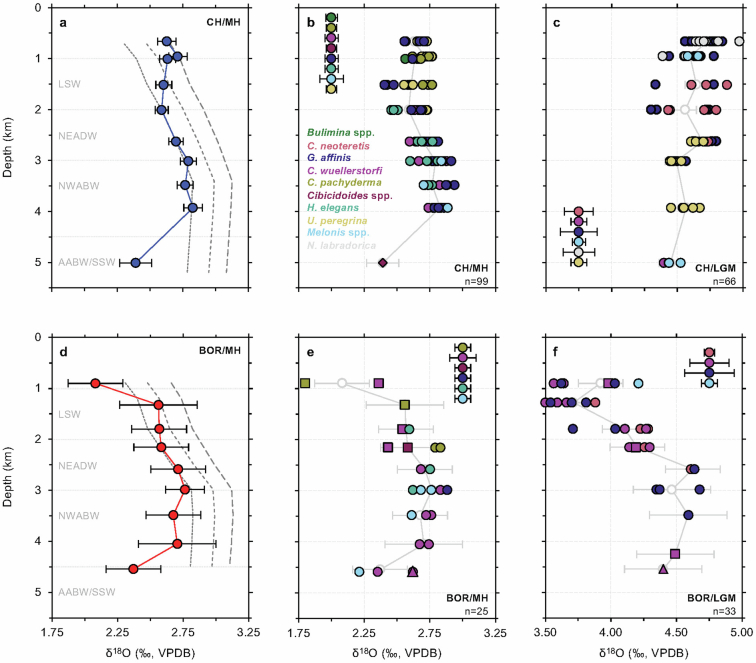
Comparison of δ^18^O profiles with modern observational data and monospecific batch data. **a**, Cape Hatteras mid-to-late Holocene δ^18^O profile (unchanged from Fig. 2) versus modern in situ-predicted δ^18^O_calcite_, which was derived using modern hydrographic observations (GLODAP v2.2022; temperature and salinity)^60^ and three different modern salinity–δ^18^O_sw_ relationships (NADW, NATL and LSW (small, medium and large dashed lines, respectively))^62,63^. To account for the influence of tilted isopycnals over the continental shelf, each profile consists of hydrographic data from the nearest GLODAP station to each core site. We note that our data show good agreement with predicted δ^18^O derived using different salinity-δ^18^O_sw_ relationships at different depths between 1 and 4.5 km, for example, sites from <1 km at Cape Hatteras are consistent with predicted δ^18^O derived using the LSW relationship. However, we also note that our new profiles are of mid-to-late Holocene age, which means that any differences between modern and mid-to-late Holocene-age δ^18^O_calcite_ could also be because of real differences in the δ^18^O composition of the ocean. Dotted lines denote the approximate depth of modern deep water mass boundaries in the North Atlantic. Mid-to-late Holocene (**b**) and LGM (**c**) monospecific batch δ^18^O data (coloured circles) with mean δ^18^O profiles (open circles and grey line). Each coloured circle denotes a repeat measurement from different mid-to-late Holocene or LGM depths, with each colour corresponding to a different species of benthic foraminifera (panel **b** lists the colour that corresponds to each species). Our new data are also supplemented by published data (squares^27^; triangle^59^; diamond^19^). Species-specific error bars are ±1*σ*, in which *σ* is the standard deviation of the species-specific reproducibility for each depth and *n* is the number of monospecific batch measurements for each transect. All infaunal δ^18^O_c_ values have been corrected to equilibrium calcite (Methods). **e**–**g**, Same as **a**–**c** but for Blake Outer Ridge. Source Data

**Extended Data Fig. 5:**
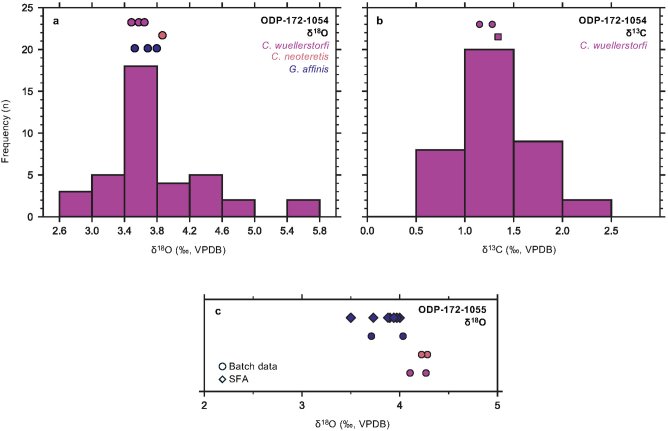
Comparison of glacial single foraminifera and monospecific batch δ^18^O and δ^13^C data from Blake Outer Ridge sites ODP-172-1054 and ODP-172-1055. **a**, Histogram of single *C. wuellerstorfi* δ^18^O measurements from sediment depths corresponding to the LGM in marine sediment core 1054. These single foraminifera data show a clearly defined peak at 3.4–3.8‰, which is in good agreement with coeval monospecific batch data (filled coloured circles). **b**, Histogram of single *C. wuellerstorfi* δ^13^C measurements from the same samples as in **a**, which show a peak between 1 and 1.5‰, which is in good agreement with monospecific batch data and published δ^13^C from nearby site KNR-140-2-56GGC (filled coloured square)^27^. **c**, Comparison of single *G. affinis* δ^18^O measurements (filled diamonds) and monospecific batch data (filled coloured circles (same as in **a**)) in marine sediment core 1055, both of which also show good agreement. In both cores, this good agreement between single and batch measurements suggests that our batch data are probably representative of in situ glacial conditions. Source Data

**Extended Data Fig. 6:**
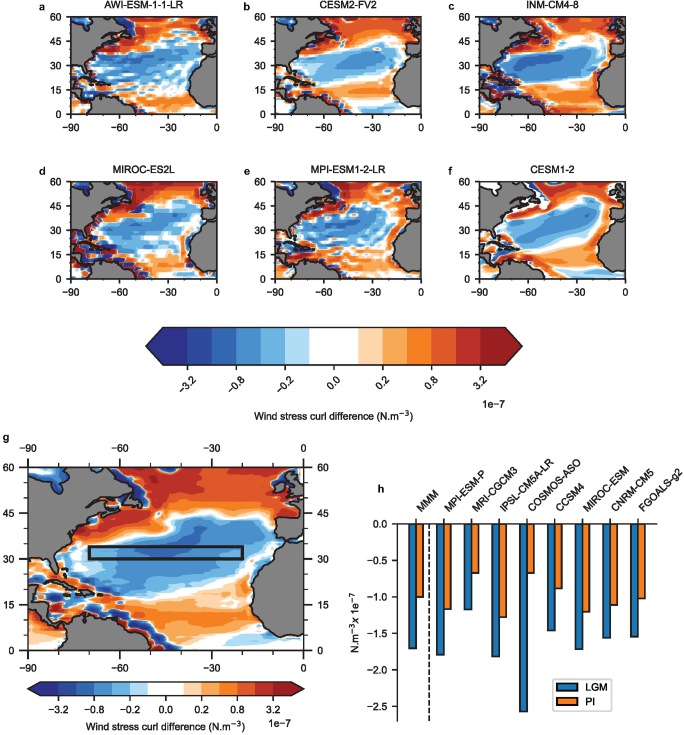
Difference between LGM and pre-industrial wind stress curl over the North Atlantic in PMIP4 and PMIP3 climate model ensembles. **a**–**f**, Individual model outputs that make up the PMIP4 MMM shown in Fig. 4. **g**, PMIP3 ensemble mean difference in wind stress curl over the North Atlantic in the LGM relative to the pre-industrial. **h**, Pre-industrial and LGM mean wind stress curl within the area shown by the black box in **g** for each of the models and the PMIP3 MMM. All eight PMIP3 models show a substantial glacial increase in wind stress curl over the STG, with an ensemble mean increase of 57% relative to the pre-industrial, which is very similar to the PMIP4 MMM (56%).

**Extended Data Fig. 7:**
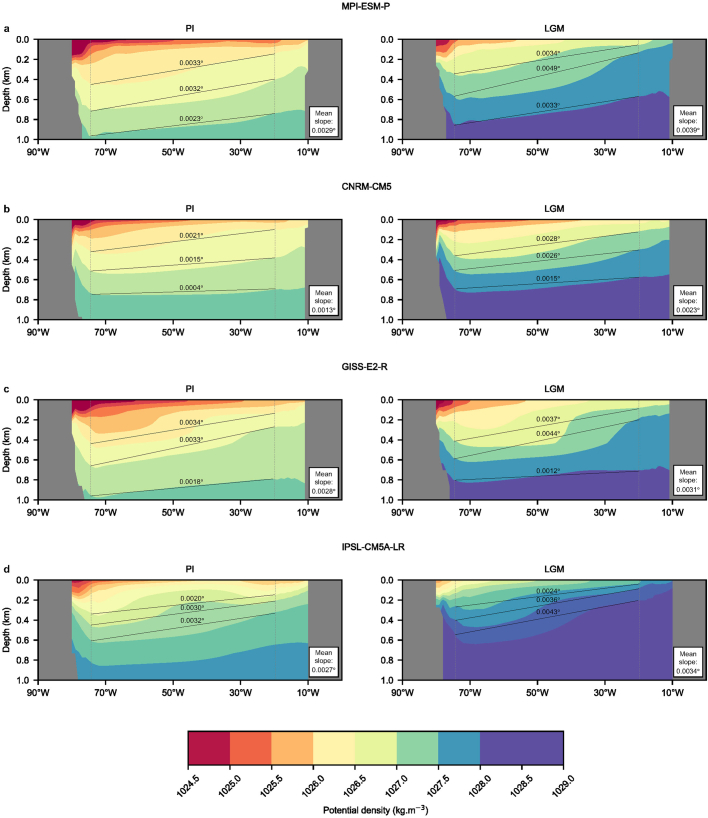
Comparison of North Atlantic potential density between 23° N and 33° N in the pre-industrial and LGM as simulated by PMIP3 climate models. **a**–**d**, PMIP3 models with available and plausible ocean data generally show a steepening of upper ocean isopycnals during the LGM relative to the pre-industrial of approximately 10–75%, consistent with proxy data and simultaneous outputs of increased glacial wind stress curl and meridional ocean velocities corresponding to the western boundary of the STG.

Previous work has raised the possibility that the reworking of some material may have occurred at shallow sites on the Carolina Slope during the LGM^27^. To test this possibility, single foraminiferal analysis was undertaken on cores ODP-172-1054 and ODP-172-1055 (Extended Data Fig. 5). Apart from one obvious outlier, δ^18^O values measured on individual specimens of *Globobulimina affinis* are tightly clustered in ODP-172-1055 (*n* = 7; ±1 s.d. = 0.17), which suggests that reworking is minimal. Single foraminifera values are also in good agreement with multispecimen (‘batch’) glacial averages; thus, we include the batch data in our glacial profiles (Extended Data Fig. 4). For ODP-172-1054, single foraminiferal analysis of *Cibicidoides wuellerstorfi* (*n* = 39) shows a clear peak at 3.4–3.8‰, which is in good agreement with our batch data and final multispecies average. Furthermore, as *C. wuellerstorfi* is a bathyal species and barely present during the Holocene at Blake Outer Ridge, we are confident that it is part of the in situ glacial population.

Most species of benthic foraminifera analysed in this study are infaunal, therefore, we have not used their δ^13^C for water mass reconstruction. Instead, we mainly rely on published compilations of epifaunal benthic foraminiferal δ^13^C that show homogenously high δ^13^C extending from about 0° to 60° N above about 2 km in the North Atlantic during the LGM^7,53^.

### Multispecies approach

To obtain robust mid-to-late Holocene and LGM δ^18^O estimates, we measured stable oxygen isotope ratios in nine different species of benthic foraminifera, before deriving a mid-to-late Holocene and LGM δ^18^O mean for each core. Because no single species of benthic foraminifera is ubiquitous spatially or temporally, a multispecies approach is also advantageous in generating measurements from cores spanning the entire subsurface water column (>0.5 km depth).

Mean mid-to-late Holocene and LGM δ^18^O values for each core were derived by first averaging monospecific batch values of the same species from different depth intervals of mid-to-late Holocene or LGM age. Typically, each monospecific mean value consists of between three and four measurements per species, but up to as many as six. We then took the average of these monospecific means to produce an overall ‘multispecies’ mid-to-late Holocene and LGM δ^18^O mean value for each core (alternative averaging methods, for example, weighting based on the number of specimens in each sample, show no notable difference). When foraminiferal abundances were low and/or bulk sediment was scarce or unavailable, we also supplemented our data with published δ^18^O data^19,27,54^ (Source Data).

### δ^18^O offsets from equilibrium calcite

Before averaging, species-specific correction factors were used to compensate for isotopic offsets from equilibrium calcite owing to metabolic variations in isotopic fractionation, that is, ‘vital effects’ (Extended Data Table 2; for infaunal species in which a range of values is published, the correction factor is determined by calculating the average offset from coeval measurements on *C. wuellerstorfi* or other epifaunal species, which, in all cases, are within the range of uncertainty of published correction factors^54,55^.

### δ^18^O compilation

To place our new δ^18^O data into a broader spatial context, we made use of previously published compilations of Holocene and glacial-age δ^18^O data from the western Atlantic, adding further data when appropriate^7,28,47–49^ (Source Data). When possible, we include mid-to-late Holocene-age data to facilitate a like-for-like comparison with our mid-to-late Holocene transect, however, published Holocene-age δ^18^O data are generally from core tops and thus probably close to modern in age. Notwithstanding, these core-top data generally show good agreement with our mid-to-late Holocene data because mid-to-late Holocene δ^18^O variability in the Northwest Atlantic is typically relatively minimal^19^. Most published δ^18^O data are also from measurements on species that calcify in equilibrium with the surrounding seawater, for example, *Cibicidoides* spp.; however, for data from species that exhibit vital effects, correction factors were applied (Extended Data Table 2).

In Fig. 2, we limited our comparison to data from sites close to both transects. For Blake Outer Ridge, this includes data from along the western boundary between Blake Outer Ridge and as far south as around 24° N, which encompasses sites at Florida Straits and the Bahamas, as well as more sites on Blake Outer Ridge and Bermuda Rise. Because there are very little published δ^18^O data from the Northwest Atlantic, we compare our Cape Hatteras transect with data from between about 36° N and 60° N. By comparison, our meridional sections (Fig. 3) include data from sites along the western boundary of the Atlantic between 55° N and 5° S.

### Sortable silt data

Samples were processed using established methods^56^ and analysed at University College London on a Beckman Coulter Multisizer 4 using the Enhanced Performance Multisizer 4 beaker and stirrer 30 to ensure full sediment suspension^57^. Two or three separate aliquots were analysed for each sample, sizing 70,000 particles per aliquot. Full procedural error based on replicates starting from newly sampled bulk sediment was ±0.32 μm (*n* = 10). Glacial mean sortable silt values were calculated by averaging data from sediments corresponding to between 19 and 23 ka.

### PMIP model outputs

We analysed LGM and pre-industrial ocean and atmosphere outputs from PMIP3 and PMIP4 models, averaging outputs over at least 100 years. Wind stress curl is calculated from the meridional and zonal wind stress components and by using a script to process CESM outputs (https://pop-tools.readthedocs.io/en/latest/examples/pop_div_curl_xr_xgcm_metrics_compare.html). For simplicity and multimodel means, other models are regridded onto the grid of CESM, which has 320 longitudes and 384 latitudes. We excluded iLOVECLIM1.1.4 from the ensemble owing to its low resolution and simplified atmosphere, which can generate substantial biases in wind stress^58^. For ocean dynamics, meridional ocean velocities between 15 °N and 40° N from 0–1.0 km depth were used, which cover the present-day latitudinal extent of the STG and are available as a direct model output. To investigate differences in the zonal gradient of the subtropical thermocline, we also analysed zonal sections of potential density spanning between 23° N and 33° N, which covers the latitudinal extent of the Sargasso Sea. We note the lack of model outputs for all oceanic variables, which is a result of the partial availability of ocean data.

## Online content

Any methods, additional references, Nature Portfolio reporting summaries, source data, extended data, supplementary information, acknowledgements, peer review information; details of author contributions and competing interests; and statements of data and code availability are available at 10.1038/s41586-024-07655-y.

### Source data

Source Data Fig. 2

Source Data Fig. 3

Source Data Extended Data Fig. 2

Source Data Extended Data Fig. 3

Source Data Extended Data Fig. 4

Source Data Extended Data Fig. 5

**Extended Data Fig. 8:**
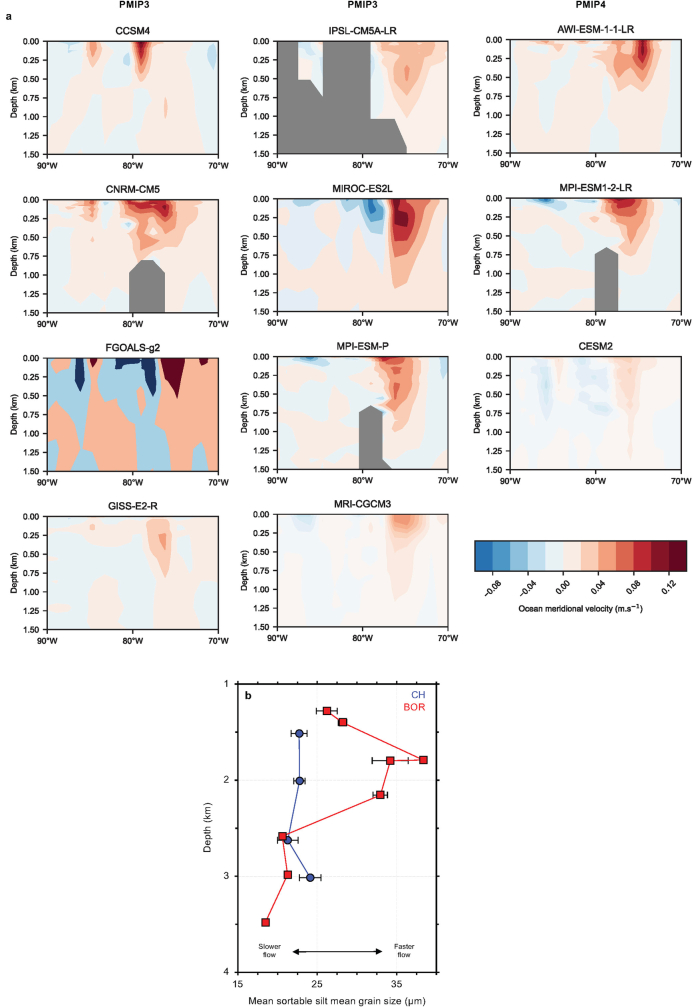
Model and proxy evidence for a more vigorous glacial Gulf Stream. **a**, Difference in meridional ocean velocities between 15° N and 40° N from 0–1.5 km depth in the LGM relative to the pre-industrial in PMIP3 and PMIP4 climate models. All models show an increase in glacial meridional ocean velocities at depths and latitudes corresponding to the Gulf Stream, which is consistent with our proxy data and other ocean and atmosphere outputs. Across the PMIP3 and PMIP4 ensembles, the LGM–pre-industrial change equates to a 44% and 51% increase, respectively, although we warn that the latter is based on only three models. **b**, New glacial sortable silt mean grain size data from core sites at Blake Outer Ridge (squares) and Cape Hatteras (circles; following previous work^33^, but using a faster stirrer speed to prevent settling^57^ (Methods)). Associated error bars are ±2 standard errors (standard error = *σ*/√*n*, in which *σ* is the standard deviation of glacial measurements for each core and *n* is the number of glacial measurements for each core). Source Data

Source Data Extended Data Fig. 8

## Figures and Tables

**Extended Data Table 1:**
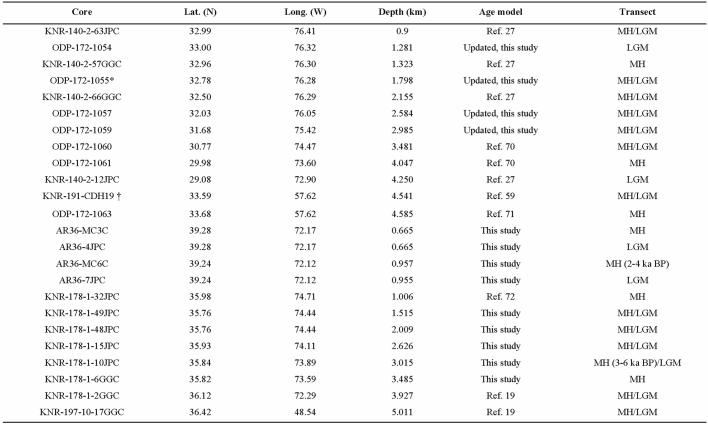
Details of cores used in this study

Refs. ^27,59^ and ^70–72^ are cited in this table.*Mid-to-late Holocene data from ODP-172-1055 are supplemented with one extra data point from nearby core KNR-140-2-51GGC^27^ (see Extended Data Fig. 4 and Source Data).

†Bermuda Rise cores KNR-191-CDH19 and ODP-172-1063 are combined to form one mid-to-late Holocene data point at roughly 4.5 km water depth.

**Extended Data Table 2:**
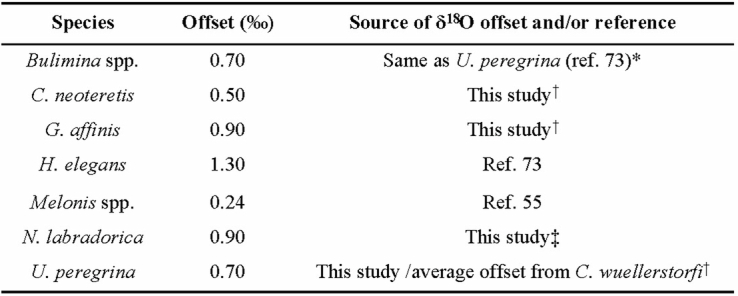
Published, unpublished and empirically derived δ^18^O offsets for infaunal benthic foraminiferal δ^18^O

*Bulimina spp. is assigned the same δ^18^O correction factor as *Uvigerina peregrina* because both species exhibit the same offset from equilibrium calcite^73^.

^†^Offsets are based on new downcore benthic δ^18^O data from Cape Hatteras cores (see Extended Data Fig. 3).

‡*Nonionellina labradorica* is assigned the same δ^18^O correction factor as *G. affinis* based on the average offset between measurements from the same samples (*n* = 3).

## Data Availability

The new proxy data that support these findings are publicly available through the data repository Pangaea at 10.1594/PANGAEA.967462. The published data^7,19,27,28,47–49,59^ used to supplement and contextualize our new δ^18^O transects can also be found in the Source Data files corresponding to Fig. 2 and Extended Data Fig. 3. GLODAP Bottle Data (version 2.2023)^60^ was downloaded from https://odv.awi.de/data/ocean/glodap-v2-2023-bottle-data/. Figures 1 and 3 and Extended Data Fig. 1 were generated using Ocean Data View software^46^, all of which feature bathymetry based on ETOPO1 global relief data^45^. Source data are provided with this paper.

## Code availability

Python code was used to analyse our proxy data and calculate wind stress curl, ocean velocities and ocean density in the PMIP3 and PMIP4 models, all of which is freely available on Zenodo (10.5281/zenodo.10955898)^61^.
